# Adaptation of balance reactions following forward perturbations in people with joint hypermobility syndrome

**DOI:** 10.1186/s12891-021-03961-y

**Published:** 2021-01-29

**Authors:** Alexander Vernon Bates, Alison McGregor, Caroline M. Alexander

**Affiliations:** 1 Department of Surgery and Cancer, Imperial College London, London, UK; 2 Department of Therapies, Imperial College Healthcare NHS Trust, Charing Cross Hospital, London, W6 8RF UK; 3 Department of Physiotherapy, Imperial College Healthcare NHS Trust, London, UK

**Keywords:** Joint hypermobility syndrome, Ehlers Danlos syndrome, Hypermobility Spectrum disorder, Balance perturbations

## Abstract

**Background:**

Joint Hypermobility Syndrome (JHS) is a Heritable Disorder of Connective tissue characterised by joint laxity and chronic widespread arthralgia. People with JHS exhibit a range of other symptoms including balance problems. To explore balance further, the objective of this study is to compare responses to forward perturbations between three groups; people who are hypermobile with (JHS) and without symptoms and people with normal flexibility.

**Methods:**

Twenty-one participants with JHS, 23 participants with Generalised Joint Hypermobility (GJH) and 22 participants who have normal flexibility (NF) stood on a platform that performed 6 sequential, sudden forward perturbations (the platform moved to the anterior to the participant). Electromyographic outcomes (EMG) and kinematics for the lower limbs were recorded using a Vicon motion capture system. Within and between group comparisons were made using Kruskal Wallis tests.

**Results:**

There were no significant differences between groups in muscle onset latency. At the 1st perturbation the group with JHS had significantly longer time-to-peak amplitude than the NF group in tibialis anterior, vastus medialis, rectus femoris, vastus lateralis, and than the GJH group in the gluteus medius. The JHS group showed significantly higher cumulative joint angle (CA) than the NF group in the hip and knee at the 1st and 2nd and 6th perturbation, and in the ankle at the 2nd perturbation. Participants with JHS had significantly higher CA than the GJH group at the in the hip and knee in the 1st and 2nd perturbation. There were no significant differences in TTR.

**Conclusions:**

The JHS group were able to normalise the timing of their muscular response in relation to control groups. They were less able to normalise joint CA, which may be indicative of impaired balance control and strength, resulting in reduced stability.

## Background

Joint Hypermobility Syndrome (JHS) is a Heritable Disorder of Connective Tissue which is considered synonymous with Ehlers Danlos Syndrome Hypermobility Type [1]. The prevailing characteristics of JHS are joint laxity and chronic pain [2], however there are many other signs and symptoms including neurophysiological involvement [3], muscle weakness [4, 5], reduced proprioception [6–8], increased reflex latency [9], joint instability [10] and impaired balance [11].

Many people have multiple hypermobile joints and most experience no detrimental effects, a condition some call Generalised Joint Hypermobility (GJH). Prevalence of GJH is common with a UK rate estimated as 18% [12]. JHS if far less common with a prevalence estimated to be 0.75–2% [13]. Importantly the prevalence of JHS within healthcare clinics is much higher; 39% in patients presenting at a UK pain clinic [14], and 45% of patients in a West-London general rheumatology clinic [15]. Recently, the classification of JHS and GJH have altered to recognise the spectrum of the disorder from an asymptomatic generalised hypermobility through to a multiple system, painful condition called hypermobile Ehlers Danlos Syndrome (hEDS) [16, 17]. In this study we use the terms JHS and GJH as the data was collected before this new classification system was published.

One of the symptoms of JHS is impaired balance, and people with JHS fall more frequently and are fearful of falling [11]. There are several studies that have found balance during quiet standing is impaired in JHS [11, 18, 19]. However, none have explored whether balance is impaired in people with GJH. Comparing people with JHS and GJH is important as it is unclear why some people who are hypermobile have problems and others do not. Without understanding the differences between these two groups, clinicians are unable to target specific dysfunctions and rely on clinical experience rather than evidence to support their patients. In addition, dynamic balance has not been explored, where the person needs to be able to respond to a perturbation. This is particularly surprising as hypermobile people tend to fall during a task rather than during quiet standing. This study aims to address this gap in knowledge by investigating how people with JHS respond to balance perturbations, which can give additional information about control of balance [20]. We aim to see if impaired balance is inherent to hypermobility or is associated solely with JHS, and therefore due to other factor(s).

We hypothesise that people with JHS will have inferior responses to balance perturbations than GJH and normal flexibility (NF) groups.

## Methods

Ethical approval was granted by NRES London-West Ethics Committee. Informed consent was obtained from all participants. The Beighton Score and Brighton Criteria were used to classify GJH and JHS participants. The Beighton Score is a 9 point score to measure joint hypermobility [13]. The Brighton Criteria is used to classify JHS; it incorporates the Beighton Score with other major and minor features of the syndrome [13]. Inclusion criteria for the JHS group was a positive classification of JHS using the Brighton Criteria. The GJH group were classified as a Beighton Score ≥ 4, and a negative classification for JHS using the Brighton Criteria. In addition both JHS and GJH groups were required to score positively for at least one knee being hypermobile. Inclusion criteria for the NF group was a Beighton Score < 4, and neither knee being hypermobile. Exclusion criteria for all groups were a history of lower limb surgery, and neurological or medical conditions not associated with JHS. GJH and NF participants were excluded if they had any lower limb pain. JHS participants were recruited from Ehlers-Danlos Support UK, The Hypermobile Syndromes Association, and patients from a London NHS Hospital. GJH and NF participants were recruited from posters displayed in the hospital and local area. To improve recruitment, we avoided jargon in our advertising posters and communications and used the term “Double Jointed” instead of “hypermobility”. We also included a sketch of the tasks required to calculate the Beighton Score to inform potential participants what we were looking for. The poster is provided in Supplementary Information.

### Testing procedure

Reflective markers were affixed to participants’ lower limbs and pelvis. We have previously described the exact marker model in a study of the reliability of hypermobile gait [21]. In brief; four markers are placed on the pelvis, at the knee on each of the tibial and femoral condyles, the lateral and medial malleolus, the calcaneus, and the head of the 1st, 2nd and 3rd metatarsals. The dominant leg was defined using a short questionnaire [22]. Eight wireless EMG modules (Myon AG, Schwarzenberg, Switzerland) and Ambu Neuroline 720 surface EMG electrodes were affixed to subjects’ on the following muscles on the dominant leg; tibialis anterior (TA), gastrocnemius (GA), gluteus medius (GM), rectus femoris (RF), vastus medius (VM), vastus lateralis (VL), bicep femoris (BF), and semitendinosus (ST). To ensure standardisation the same researcher placed markers and electrodes on all participants and used the SENIAM guidelines for electrode placement, orientation, starting position and clinical test to ensure correct placement [23]. In addition, training on surface anatomy and the first sessions were observed by an experienced clinical physiotherapist (CMA – 32 years experience). Trials were recorded using a 10 camera Vicon (Oxford Metrics Ltd., Oxford, UK) which synchronised kinematic and EMG data. Kinematic data was sampled at 100 Hz, EMG data at 1000 Hz.

A custom platform (120 cm × 120 cm) was developed capable of producing perturbations in four directions. Only forward perturbations (where the platform moves to the anterior of the participant) were used as they have been reported by participants to be most difficult [24] which we confirmed in pilot testing. An accelerometer (sampling at 1000 Hz) was attached to the platform and used to determine perturbation start times. It was synchronised to EMG and kinematic data using the Vicon system.

Participants rated their knee pain using a visual analogue scale (VAS). Participants were asked to stand on the platform with their feet hip-width apart, arms relaxed by their sides, looking straight-ahead with eyes open. The position of the feet was marked; if a participant needed to take a step to balance they were asked to return to the same starting position. Participants wore a shoulder harness attached to the ceiling and had parallel bars available as a safety precaution. Participants reported that neither shoulder harness or bars impeded movement. Participants were told that the platform would move forwards but they were not told the timing of perturbations to limit anticipatory responses. Six perturbations were performed, with approximately 3–5 s of rest between each perturbation. The translation distance was 4.0 cm, time 0.2 s. The perturbation consisted of an acceleration to peak velocity at 0.1 s, followed by a deceleration phase to stopping.

### Data processing and analysis

**Fig. 1:**
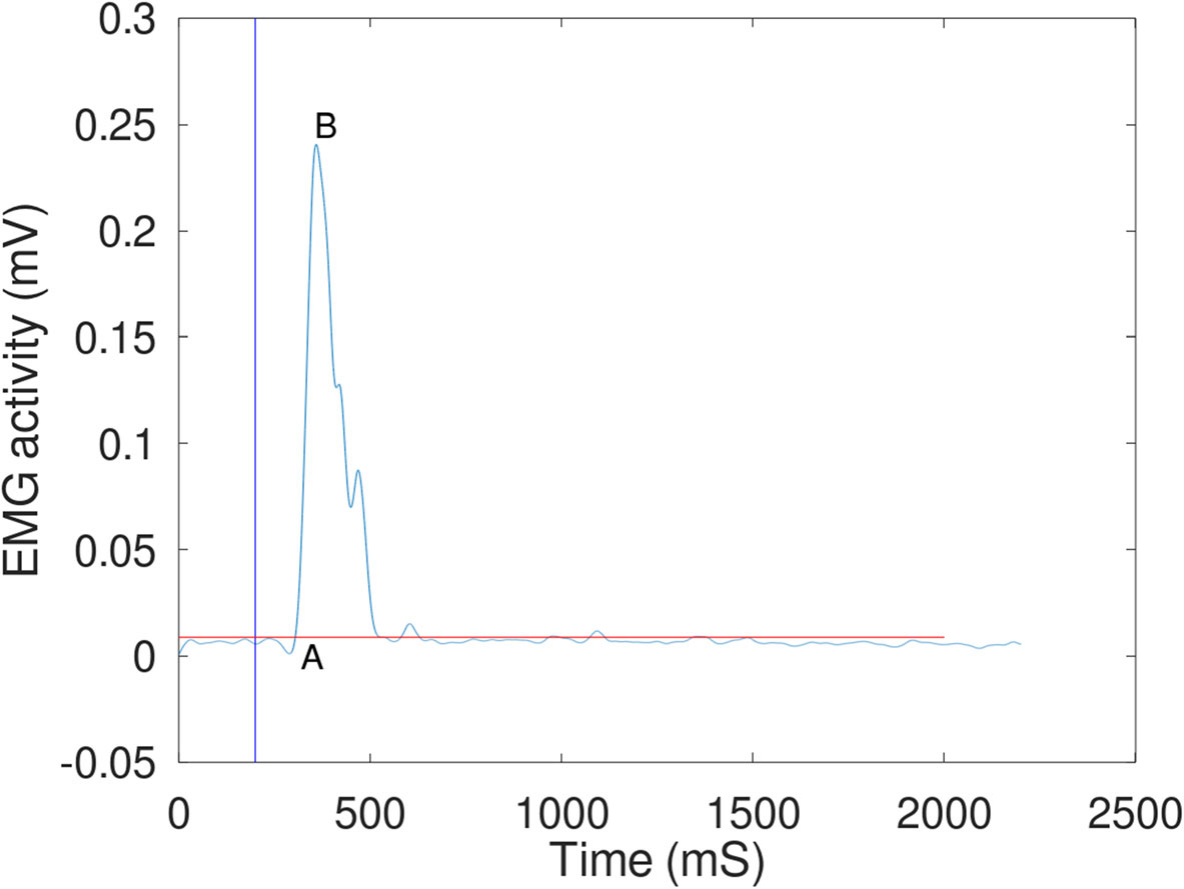
An example of processed EMG data from a participant with Joint Hypermobility Syndrome. Data from the tibialis anterior muscle. Blue vertical line is the perturbation start time, red horizontal line is the activation threshold. Outcome measures were time-to-activation; the time from perturbation start to muscle activating (point **a**), and time-to-peak; the time from activation to reaching peak intensity (the time from point **a** to point **b**)

EMG, kinematic and accelerometer data was processed in MatLab. Perturbation onset was defined as the time the perturbation platform reached 5% of its peak acceleration. EMG data was band-pass filtered between 20 and 500 Hz, full-wave rectified and low-pass filtered at 15 Hz. A muscle was considered active if its EMG amplitude was 2 standard deviations above the pre-stimulus mean, which was calculated during the 200 ms before the perturbation. EMG variables analysed were onset latency (time taken for a muscle to become active following perturbation onset) and time-to-peak amplitude (time taken for a muscle to reach peak EMG amplitude following perturbation onset). A muscle was considered active if its EMG amplitude was 2 standard deviations above the pre-stimulus mean, which was calculated during the 200 ms before the perturbation. See Fig. 1 for an example of EMG data. We were limited in what EMG variables could be collected as pilot experiments revealed that reliable maximal voluntary contractions (MVC) from the JHS group could not be elicited. In the group with JHS EMG amplitude recorded during a perturbation was up to three times greater than that recorded during an MVC. Therefore, we were unable to normalise EMG data with respect to amplitude and so were unable to compare peak voltages between groups. We therefore limited our measurements to the temporal EMG variables described above.

**Fig. 2:**
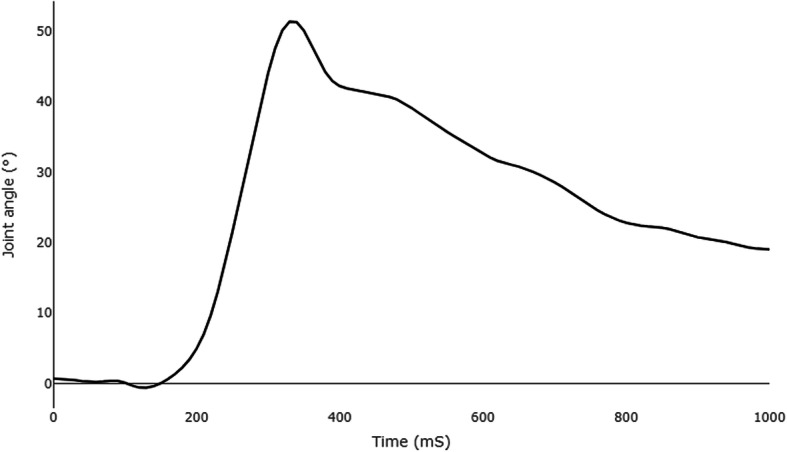
Example of knee joint angle during a perturbation of a participant with JHS. Perturbation occurred at 0 mS. The Cumulative Angle (CA) outcome measure is the sum of all absolute changes throughout the perturbation

Kinematic data was low-pass filtered at 10 Hz. Kinematic variables analysed were the cumulative sum of change in angle (CA) and time-to-reversal (TTR). A commonly analysed outcome in studies of balance perturbations is maximum joint excursion. CA differs in that it is the sum of the absolute changes in angle of a joint. For example, if a joint was to ‘wobble’ maximum excursion would only identify the maximum value, whereas CA represents the total amount of movement in a joint (see Fig. 2). TTR is the time taken to reverse the initial change in angle from perturbation onset for ankle dorsiflexion/platarflexion, knee extension/flexion and hip extension/flexion. Both CA and TTR were analysed from perturbation onset to 1 s post perturbation. The dominant limb was used for analysis. A recovery step was performed if a participant moved either foot within 1 s of the perturbation start.

Typically in studies of postural responses following perturbation, the greatest adaptation is found between the 1st and 2nd perturbation (P1 and P2). To investigate how each cohort changed their behaviour to perturbation, we made within-group comparisons between the 1st and 2nd perturbation to measure initial adaptations, and between the 2nd and 6th perturbations to determine if groups continued to adapt their response. Similarly, between-group comparisons were made at the 1st, 2nd and 6th perturbation.

### Statistical analysis

Sample size was informed by the variability of kinematics of hypermobile movement reported in a previous reliability study [21], indicating a minimum sample size of 20 participants in each group were required to reach sufficient power where *p* = 0.05 (β = 0.2). Statistical analysis was performed using IBM SPSS version 24. A Sharipo-Wilk test was used to assess variables for normality. Tests within and between group were explored with Kruskal Wallis tests for non-normally distributed data and MANOVAs were used for normally distributed data. Significant differences were explored with pair-wise comparisons and a Bonferroni correction was applied to correct for multiple comparisons.

## Results

Twenty-three people with JHS, 23 people with GJH, and 22 NF people were recruited. Two JHS participants chose not to participate in the perturbation task; one due to fear of stepping up onto the perturbation platform, and one did not proceed beyond the 3rd perturbation due to anxiety about falling. Their results have been omitted from this analysis. There were no adverse events and no participants fell or used the hand-rails following a perturbation. Participant characteristics are shown in Table 1. As expected, the NF group had a lower Beighton score than the JHS (*p* < .001). There were no other significant differences between groups.

**Table 1:** Participant characteristics (mean ± SD)

	JHS (*n* = 21)	GJH (*n* = 23)	NF (*n* = 22)
Age (years)	33 ± 9	28 ± 6	28 ± 5
Sex (f/m)	18/3	19/4	16/6
Height (cm)	169 ± 8	169 ± 10	172 ± 8
BMI	25.5 ± 5.6	22.9 ± 4.4	22.0 ± 2.8
Beighton Score ^a,b^	6.8 ± 2.1	6.6 ± 1.3	0.3 ± 0.7
Visual analogue score knee pain, scored from 0 to 10	3.2 ± 2.2	0.0 ± 0.0	0.0 ± 0.0

^a^significant difference between JHS and NF

^b^significant difference between GJH and NF groups

Significance after Bonferroni corrections set at *P* < .05

A greater proportion of JHS participants took a recovery step on the 1st perturbation (visual inspection of the data precluded the need for statistical analysis). Only two JHS participants performed a recovery step from P2 onwards; one performed steps up to P4, and one who performed a step on every perturbation. One GJH participant consistently performed a recovery step, and one NF participant performed a recovery step on P1. All results are included regardless of whether a recovery step was taken.

The EMG results of muscle onset and time-to-peak amplitude were not normally distributed; medians and quartiles are shown in Table 2. There were no significant differences in muscle onset times between groups at any perturbation number. However, there were significant differences for time-to-peak amplitude at P1 for TA, GM, RF, VM and VL. JHS time-to-peak was significantly later than the NF group in TA (*p* = .020), RF (*p* = .002), VM (*p* = .011), and VL (p = .002), and significantly later than GJH in GM (*p* = .001) and VL (*p* = .008). There were no significant differences in these parameters between JHS and NF groups for P2 and P6 perturbations; the JHS group time-to-peak amplitude had reduced such that it was similar to NF in most muscles. However, at P6 the later time-to-peak amplitude persisted in GM (*p* = .003) between JHS and GJH groups (*p* = .007). There was no significant difference between GJH and NF groups at any perturbation.

**Table 2:** Median (1st quartile, 3rd quartile) for EMG parameters. Onset and TTP amplitude are in milliseconds. *TA* Tibialis anterior, *GM* Gluteus medius, RF Rectus femoris, VM Vastus medialis, VL Vastus lateralis, BF Biceps femoris, ST Semitendinosus. P*(i)* = perturbation number. ^a^significant difference between JHS and GJH, ^b^significant difference between JHS and NF. Significance set at *P* < =.05

		P1			P2			P6		
		JHS	GJH	NF	JHS	GJH	NF	JHS	GJH	NF
TA	Onset (ms)	88 (79.3,96)	86 (76,99)	88 (82,99)	89 (80.8,93.5)	88.5 (75,97)	87.5 (76,103)	88 (79.5,94.3)	84 (73,91)	79 (76,89)
	TTP (ms)	**175 (119,298.5)**^**b**^	119 (72,152)	**101 (74,135)** ^**b**^	103 (85.5149.3)	99 (75,138)	89 (62,117)	108 (83.8134.8)	93.5 (73,121)	102 (82,119)
GM	Onset (ms)	130 (113.5148.5)	116 (101,135)	130.5 (111,148)	131 (101.3154.3)	113 (85,135)	128 (114,153)	134 (68.5151)	128 (89,147)	129 (105,152)
	TTP (ms)	**167 (103.8226.5)**^**a**^	**78 (63,84)**^**a**^	93.5 (68,129)	89 (75,149.8)	79.5 (35,101)	70.5 (56,89)	**89 (45.8136.5)**^**a**^	**51.5 (22,82)**^**a**^	60 (30,71)
RF	Onset (ms)	117 (108,123.5)	110 (100,118)	119 (102,126)	116 (99,130.3)	110 (88,121)	113.5 (102,126)	107 (87,124.3)	108 (96,118)	112 (97,121)
	TTP (ms)	**153 (109.5196.5)**^**b**^	99 (81,140)	**82 (75,107)** ^**b**^	115 (84,231.3)	86 (69,99)	93 (74,104)	81 (59.5140.3)	80.5 (70,94)	79 (71,89)
VM	Onset (ms)	121 (110,128.5)	118.5 (105,141)	126 (109,147)	118 (99.5136)	116 (100,149)	114.5 (97,133)	123 (92.3129.3)	115.5 (94,147)	115.5 (90,137)
	TTP (ms)	**133 (77,240)**^**b**^	117.5 (83,150)	**85.5 (68,100)** ^**b**^	93 (61.8196)	88 (74,114)	70 (45,101)	78 (46.3211.8)	70 (56,99)	56 (25,85)
VL	Onset (ms)	114 (109.3132)	106.5 (94,114)	112.5 (102,123)	116 (103.5137.8)	109.5 (89,127)	116 (98,126)	116 (99.8123.5)	99.5 (92,127)	104 (91,120)
	TTP (ms)	**193 (114.5292.3)**^**b**^	90 (73,125)	**95 (77,122)**^**b**^	140 (85,192.3)	88.5 (72,112)	88 (72,101)	79 (61.5147.5)	76 (66,84)	82.5 (68,93)
BF	Onset (ms)	106 (97.8119.8)	95.5 (82,121)	108 (82,134)	110 (103,120)	96 (78,117)	103 (88,128)	106 (92.5141)	104.5 (83,123)	102.5 (66,134)
	TTP (ms)	105 (82.8219.8)	100 (61,167)	99.5 (69,150)	127 (81.5156.3)	89 (66,123)	102 (84,148)	69 (53.8101.5)	70.5 (45,87)	77 (53,88)
ST	Onset (ms)	99 (92.8111.3)	91 (84,115)	92.5 (75,153)	110 (88.3128.3)	91.5 (71,110)	105.5 (63,134)	120 (84.3141)	112.5 (86,133)	86 (59,142)
	TTP (ms)	86 (69.3151)	91 (66,194)	100 (72,163)	98 (72,129.3)	85 (56,131)	81.5 (57,119)	67 (40,83)	61.5 (44,84)	82.5 (39,104)

Moving to kinematic results, TTR results are shows in Table 3; there were no significant differences for both within and between group comparisons.

**Table 3:** Time-to-reversal mean ± standard deviation (milliseconds). There were no significant differences between groups or between perturbations

		JHS (*n* = 21)	GJH (*n* = 23)	NF (*n* = 22)
P1	Hip	270 ± 80	260 ± 80	280 ± 70
	Knee	310 ± 70	290 ± 50	270 ± 40
	Ankle	320 ± 50	320 ± 60	310 ± 80
P2	Hip	270 ± 50	270 ± 50	290 ± 130
	Knee	270 ± 60	300 ± 60	260 ± 50
	Ankle	270 ± 60	300 ± 60	320 ± 90
P6	Hip	270 ± 60	270 ± 70	270 ± 120
	Knee	300 ± 50	290 ± 50	280 ± 70
	Ankle	320 ± 110	320 ± 70	330 ± 120

**Fig. 3:**
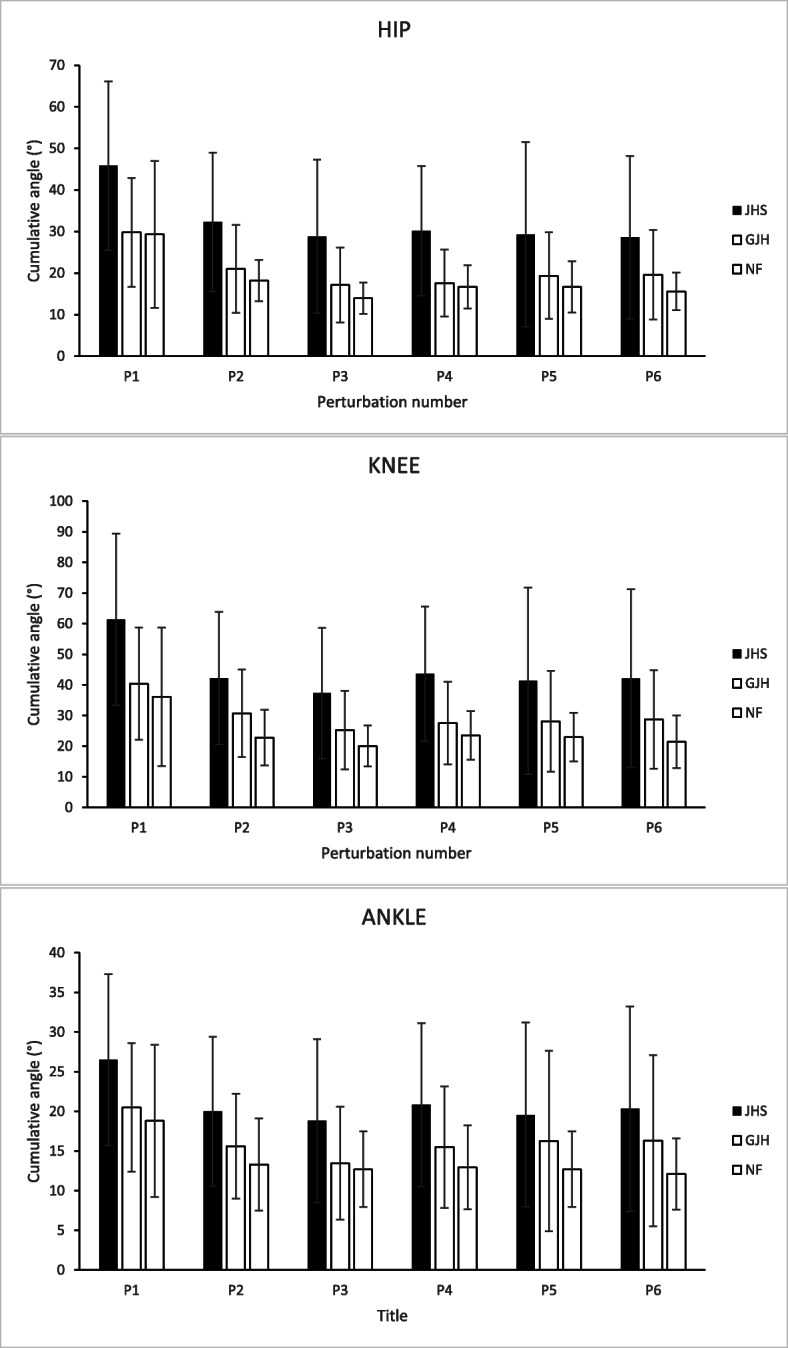
Cumulative angle following perturbation for the hip, knee and ankle joints. NF is Normal Flexibility, GJH is Generalised Joint Hypermobility, JHS is Joint Hypermobility Syndrome. P*(i)* = perturbation number

P1 elicited the greatest magnitude of cumulative angle (CA) in all groups around all joints (see Table 4 and Fig. 3). For between group comparisons, generally the group with JHS had significantly greater CA than GJH and NF groups. At P1 the group with JHS had significantly greater CA than GJH and NF groups at the hip (*p* = 0.041 and *p* = 0.042 respectively) and knee (*p* = 0.035 and *p* = 0.009 respectively). At P2 the group with JHS had significantly greater CA than GJH and NF groups at the hip (p = 0.03 and *p* < 0.01) and knee (*p* = 0.028 and p < 0.01 respectively), and greater CA at the ankle than NF (p < 0.01). At the final perturbation (P6), the only significant differences were between the group with JHS and NF group, with the group with JHS showing greater hip and knee CA (*p* = 0.018 and p < 0.01). There were no significant differences in CA between GJH and NF groups at any perturbation number.

**Table 4:** Mean cumulative joint angles ± standard deviation. ^a^ significant difference between JHS and GJH, ^b^significant difference between JHS and NF. Significance after Bonferroni corrections set at *P* < .05

		JHS(*n* = 21)	GJH(*n* = 23)	NF(*n* = 22)
P1	Hip ^a,b^	45.8 ± 20.3°	29.8 ± 13.1°	29.3 ± 17.7°
	Knee ^a,b^	61.4 ± 28.0°	40.4 ± 18.3°	36.1 ± 22.6°
	Ankle	26.5 ± 10.8°	20.5 ± 8.1°	18.8 ± 9.6°
P2	Hip ^a,b^	32.3 ± 16.7°	21.0 ± 10.6°	18.2 ± 5.0°
	Knee ^a,b^	42.2 ± 21.6°	30.7 ± 14.3°	22.8 ± 9.1°
	Ankle ^b^	20.0 ± 9.4°	15.6 ± 6.6°	13.3 ± 5.8°
P6	Hip ^b^	28.6 ± 19.6°	19.6 ± 10.8°	15.6 ± 4.5°
	Knee ^b^	42.1 ± 29.1°	28.7 ± 16.1°	21.4 ± 8.6°
	Ankle	20.3 ± 12.9°	16.3 ± 10.8°	12.1 ± 4.5°

For within-group comparisons, in all groups the greatest difference between perturbations was between P1 and P2; in all groups hip, knee, and ankle CA was significantly lower at P2 than at P1. *P*-values for the hip, knee, and ankle were *p* = 0.022, p < 0.01 and p < 0.01 in the JHS group; *p* = 0.033, *p* = 0.037 and p < 0.01 in the GJH group; and p < 0.01, p < 0.01, and p < 0.01 in the NF group. Differences between subsequent perturbations were relatively smaller, and the only significant difference between P2 and P6 was in JHS hip CA, which was lower at P6 (*p* = 0.011).

## Discussion

This is the first study to demonstrate the differences between JHS, GJH and NF groups in their responses to a perturbation. Generally, the JHS group had a greater cumulative angle during perturbations than the other groups; the EMG did not differ in the same way.

To stop a fall following a perturbation a person must retain their centre of mass over their base of support. This can be achieved through muscular contractions alone and keeping the same base of support, or by taking a step and increasing the base of support area [24]. In this study more JHS individuals took a recovery step at P1 than control groups, indicating that they either have impaired ability to perform the muscular response necessary to counter the perturbation, or they are more anxious of falling and take a step ‘just in case’. Although a high proportion of JHS participants took a recovery step at P1, only one JHS participant took a recovery step for P2-P6. This shows that JHS participants were able to ‘learn’ to improve their postural response or alternatively were less afraid of falling and so deemed a recovery step unnecessary.

Moving to EMG results; in general there was not a consistent pattern of how the group with JHS differed to control groups. This could be due to several factors. Firstly the results are variable; it is possible participants may be using different strategies to address the forward perturbations [25], thus increasing the within-group variability and making differences more difficult to observe. Secondly, taking a broad approach most of the significant differences were observed between the group with JHS and the group with NF, and only several differences observed between the group with JHS and the group with GJH. The sample size is based on kinematics of hypermobility (see ‘Limitations’) and may not be fully powered to uncover any smaller differences between groups. Despite the variability, our results conform with those of other studies in that postural reactions occurred between 70 and 180 ms post perturbation [25, 26]. Studies of perturbation response in other patient cohorts have found differences in muscle onset timing [24, 27]^,^ that were not observed here. [24, 28] Despite people with JHS having reduced proprioception [7, 8] and deficiencies in the neurophysiological system [9], they were able to initiate a muscular response with similar timing as controls. This indicates that these mechanisms are not impaired to a degree that would affect the ability of JHS participants to initiate a timely muscular response. However, for TTP parameters at P1 the JHS group took significantly longer than the NF group to reach peak muscle amplitude in TA and quadriceps muscles, and in the GM muscle when compared to the GJH group. This may explain differences in CA between JHS and NF/GJH groups at P1; JHS participants are taking longer to produce a peak force and so are less able to arrest the momentum of the centre of mass.

Due to the multifactorial nature of JHS there are many mechanisms that may contribute to these results. One important factor could be that the group with JHS were in pain. Given that all participants with JHS had knee pain and GJH and NF participants were pain free, it could be that pain is affecting postural responses. Rombaut et al. [11] highlighted mechanisms of how pain could limit a person’s response to perturbations, namely by increasing reflex inhibition around the knee joint [29], and limiting their ability to respond by reducing loading in painful joints. The pain may be one factor in driving some of the differences seen here; however, the pain didn’t adapt with the perturbations therefore it seems unlikely to be the driver for the adaptations seen here. An alternative potential explanation is adaptation to hypertonia, which may be one of the features of JHS^35^. This could be peripherally or centrally driven. For example, in relation to peripherally driven factors, a longer TTP could be explained by muscle receptors sitting within more flexible connective tissue and hypotonic muscle. So, for example, the muscle spindle may not be as responsive to changes in length, and consequently afferent feedback to the central nervous system may be less effective resulting in the muscle taking longer to reach the peak that the other groups attained. The centrally mediated anticipatory control can alter postural responses [20], and JHS participants might increase the tension in their muscles from P2 onwards as a way of adapting to lower muscle tone and/or weakness. This would explain why there were no significant differences in TTP amplitude at P2 and P6. However, the mechanisms behind the differences seen here were not explored therefore, any explanation is speculative. Delays in TTP amplitude have been observed in an elderly cohort and De Freitas et al. [24] proposed several other mechanisms; a reduction in the ratio of Type II to Type I muscle fibres, differences in motor unit discharge frequency, and/or a reduction in doublet discharges. Although the results here may indicate that JHS muscular responses are impaired, they do not allow for a more nuanced investigation of the underlying cause.

Except for GM at P6, the JHS group could match their TTP amplitude response to the other groups from P2 onwards. Importantly, whilst the time-to-peak parameters of the JHS group normalised, this did not translate to normalisation of CA parameters. At each perturbation measured, the JHS group had consistently higher CA than NF and GJH in all joints (Fig. 3), which may indicate that their movement control was deficient and remained more unstable than NF and GJH groups. In all groups, P1 produced the greatest magnitude of CA. All groups significantly reduced their CA in all the joints measured between P1 and P2, showing that all groups adapted and improved their postural response. For NF and GJH groups, there was no significant difference in any joint between P2 and P6, indicating that there is little or no further adaptation to the perturbation. In the JHS group, hip CA at P2 was significantly greater than P6, which may mean that they are slower, or unable, to adapt their responses as quickly as the two control groups.

Figure 3 illustrates that the GJH CA lies consistently between JHS and NF for all joints at all perturbations. Although differences between GJH and NF were not significant, the fact that the GJH group had consistently greater CA values than NF may indicate that hypermobility has a contributory effect on instability. However, since no significant differences between GJH and NF groups were recorded in any joint at any perturbation, it seems unlikely that joint hypermobility per se is responsible for the increased instability, but rather another factor of JHS that is not present in GJH. The wide range of symptoms of JHS makes it difficult to pinpoint a specific reason. There is evidence that there are neurophysiological differences in people with JHS; differences have been shown in proprioception [6–8], autonomic dysfunction [3], and in musculoskeletal reflexes [9]. Reduced proprioception could cause JHS participants to be less able to sense the magnitude of the perturbation and/or the position of the joints that result in a stable base of support. Alternatively, the reason for increased instability could be due to muscle weakness [4, 5], which would limit the ability to control the inertia and momentum produced by the perturbation.

## Conclusions

Overall the greater muscle TTP amplitude during P1, and greater CA translates into greater instability in the JHS group. This may explain why JHS individuals fall and have a fear of falling [11]. The results of this study raise the question whether perturbation-based training and or strengthening could help improve balance. Training would seem a promising strategy as there are several strategies for addressing forward perturbations, which are not hard-wired but can be learned [25]. Furthermore, perturbation training has produced improvements in other cohorts [30–33]. However, if the differences seen here relate to muscle strength alone, then a strength programme may be more effective. A direction for future research would be to test if perturbation training or strength training would improve JHS perturbation responses and subsequently frequency of falls and falls risk.

### Limitations

Participation bias may have been a factor in our recruitment. It is feasible that JHS individuals whose symptoms are more severe, would avoid a study based on movement which they perceive may cause discomfort. We were not able to obtain reliable MVCs from JHS participants. Although pain may have been a limiting factor in producing MVCs, participants reported that pain was not limiting their effort during the MVC procedure. Future work would benefit by normalising EMG to the amplitude of evoked potentials. The power of this study was based on previous analysis of the variability of kinematic measures in people with hypermobility. This power calculation may not fully capture all variables explored (particularly EMG variables). It may be that more nuanced differences between groups would be identified with a greater sample. This study was completed prior to the newer classification criteria and it was not possible to retrospectively classify participants as hypermobile EDS/Hypermobility Spectrum Disorder. However, it is interesting that differences were still observed without the benefit of the stricter classification criteria.

## Data Availability

The datasets used and/or analysed during the current study are available from the corresponding author on reasonable request.

## Abbreviations

BF: Bicep femoris
CA: Cumulative angle
EMG: Electromyography
GA: Gastrocnemius
GJH: Generalised Joint Hypermobility
GM: Gluteus medius
JHS: Joint Hypermobility Syndrome
MVC: Maximal voluntary contraction
NF: Normal Flexibility
RF: Rectus femoris
ST: Semitendinosus
TA: Tibialis anterior
TTR: Time to reversal
VAS: Visual analogue scale
VL: Vastus lateralis
VM: Vastus medius
